# Shear Testing of Topologically Optimised Web Cover Plates in Splice Connections—Experiment Design and Results

**DOI:** 10.3390/ma16227077

**Published:** 2023-11-08

**Authors:** Tiago Ribeiro, Luís Bernardo, Miguel C. S. Nepomuceno, Natale Antonio Maugeri, Paolo Longo, Dario De Domenico

**Affiliations:** 1 Department of Civil Engineering and Architecture, University of Beira Interior, 6201-001 Covilhã, Portugal; 2 Centre of Materials and Building Technologies, Department of Civil Engineering and Architecture, University of Beira Interior, 6201-001 Covilhã, Portugal; 3 Department of Engineering, University of Messina, 98166 Messina, Italy

**Keywords:** topology optimisation, splice connection, shear, ultimate capacity, buckling, experimental results

## Abstract

Testing shear-resisting plates in steel connections is one of the most challenging laboratory undertakings in steel construction, as the most common experimental layout design includes simulating the connection with its adjoining members. This significant hindrance gained particular magnitude as the need to test prototypes of topologically optimised shear cover plates became more pressing. Indeed, new code-compliant topology optimisation approaches for steel construction have recently been offered, and physically non-linear analyses have been demonstrated to be vital for assessing these elements. Hence, a rapid and reliable experimental process has become a fundamental necessity. To answer this need, a novel layout is herein proposed, in which topologically optimised and previously numerically examined bolted shear plates of a well-known steel joint were tested. The results allowed for the definition of the material trilinear model for use in subsequent numerical analysis, as well as the validation of the numerical simulation results. The discrepancy between the previously mathematically anticipated and empirically determined ultimate resistance did not exceed 1.7%.

## 1. Introduction

Shear testing of steel bolted connection parts has always been challenging for laboratory researchers [1,2,3,4]. The fact that these connections’ centre of rotation depends on the number and position of the bolts (changing as these undergo non-linearity) and that cover plate connections are made of several independent parts makes it difficult to design an experimental test, except if specific machinery is available.

In the current case, the need to test exceptionally slender shear parts and the availability of a general-purpose tension testing machine led to the design of a novel testing apparatus that can be employed in several other cases.

The experimental programme aims to assess the ultimate and buckling behaviour of topologically optimised web cover plates [5,6] originally designed for the seminal work of Sheikh-Ibrahim on steel girder splice connections [7,8,9].

Experimental validation has been considered paramount for leveraging topology optimisation (TO) for steel construction after recent developments on the proposition and numerical validation of a methodology for code-compliant TO for steel connections [10]. Moreover, the same research initiative also showed that the behaviour of topologically optimised bolted connections cannot be safely modelled in the linear regimen [10], meaning that non-linear analyses shall be conducted, thus imposing a further need for experimental validation.

Hence, the current study is framed within a comprehensive research programme deemed to find a much-needed solution to bring TO to practice in steel construction and fill an important gap in relation to other industries where TO is already a daily reality [11].

Prototypes were optimised using a solid isotropic material with a penalisation (SIMP)-based approach using the TOSCA software (version 2017) following a Eurocode 3-compliant methodology.

The literature was investigated for state-of-the-art experimental setups for shear testing. The works of Chen et al. for shear tests on cold-formed steel channels [12], Milewska et al. on proposing design rules for shear testing [13], Lu et al. on experimental investigation of the shear behaviour of connectors [14], and Fan et al. [15] and Cai et Yuan [16] on the shear behaviour of steel connections have been considered.

In light of the depicted literature, one can frame the current experimental research within the field as a contribution to a better understanding of the shear behaviour of highly optimised shear parts.

The main aims of the current document are reporting a novel layout for rapid and reliable shear testing of shear cover plates, as well as experimentally determining the ultimate behaviour of topologically optimised shear plates in a way that previous results from numerical non-linear analyses can be validated. The results herein presented to the community are extensive with a further aim of enabling full reproducibility and sustaining the deduction of critical conclusions from previous numerical calculations.

## 2. Materials

### 2.1. Sheikh-Ibrahim’s Splice Connection

#### 2.1.1. Geometry

Sheikh-Ibrahim’s work at the University of Texas at Austin in 1995 [7] provided a wide-ranging comprehension of moment connections in steel beams, particularly concerning the effects of bolt row eccentricity on web plates, thus contributing to the development of design recommendations that are broadly used by engineers and researchers [8,9].

**Figure 1:**
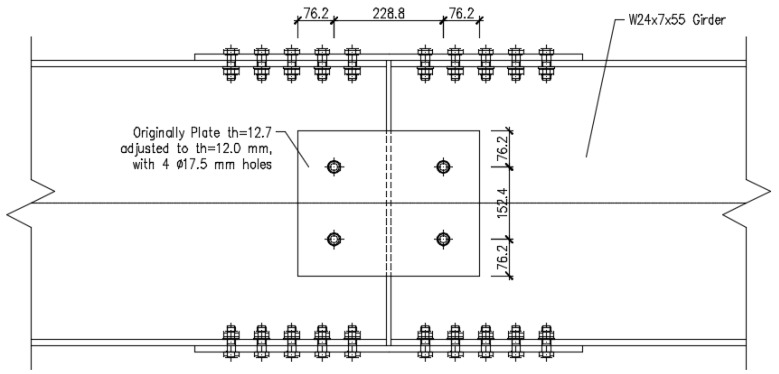
Web connection layout (dimensions in mm).

This scenario, together with the abundance of experimental data available, made it appropriate to examine one of the examples analysed for implementing a novel TO approach. As a result, Sheikh-Ibrahim’s first case (1 of 32) was investigated, as shown in Figure 1 in which two W24 × 7 × 55 segments in A36 steel [17] are coupled with one cover plate in each flange and two cover plates in the web. The web cover plates are 381.2 mm in length, 304.8 mm in width, and 12.7 mm thick. Because the fabrication was performed in Europe, the plate thickness was reduced to 12 mm. Similarly, the original A325 steel bolts with 15.9 mm diameter were replaced with M16 (8.8) bolts.

#### 2.1.2. Connection and Plate Resistance

The European steel construction standards [18,19,20] were used to establish an initial scenario for the predicted failure modes of the non-optimised plate. Within this context, it was determined that the connections’ capacity is restricted by their bending moment resistance, which was calculated to be 417 kNm and 514 kNm depending on whether or not the partial safety coefficients relevant to the ultimate limit states were included. Given the characteristic resistance of the connection (417 kNm), the related shear when the connection fails in bending is 151 kN.

Each 12.0 mm thick web cover plate has a bearing resistance of F_bRd_ = 0.00048 f_u_ [kN] (with fu in kPa and no partial safety factor of γ_M2_ = 1.25). According to EN1993-1-8, each M16 class 8.8 bolt has a shear resistance of F_vRd_ = 75.4 kN (without the partial safety factor of γ_M2_ = 1.25). As a result, the bolt shear limits the plate capacity rather than its bearing capacity, which equals 192 kN for the standard’s minimal threshold for ultimate stress.

In addition to the connection’s vertical shearing forces, the inherent eccentricity of the bolt rows leads to a bending moment opposed to a torque with horizontal components [7]. For R_,vector_ = 1 (unitary resultant force), this leads to vertical and horizontal components in the bolts equal to V_,component_ = 0.555 and H_,component_ = 0.832, respectively, indicating that each bolt capacity is 75.4 kN (per bolt and shear plane). The joint shear resistance is four times the vertical component. Thus, V/4 = 0.555 × 75.4, and the connection’s resistance to an externally induced shear force is V = 167 kN.

### 2.2. Topology Optimisation

The web plate of the connection was topologically optimised utilising a density-based technique, the solid isotropic microstructure with penalisation (SIMP) method [11], with a penalty factor of 3. Svanberg’s method of moving asymptotes (MMA) solver was utilised [21,22].

Following a methodology proposed in [6], a Eurocode-compliant procedure was implemented, ensuring that the relevant collapse modes are considered by means of geometric constraints. The optimisation computational models employed the finite element method with two-dimensional triangular quadratic elements (STRI65) on free meshes with partitions. The selected objective function was the energy stiffness measure minimisation.

To that goal, parametric research was conducted, resulting in optimal topologies with variable volume fractions from 100% to 10% every 10% in the first stage and a refinement every 2.5% in the second stage, from which physically non-linear studies were able to determine the precise final capacity of each solution. The selected topology corresponds to the smallest volume percentage that demonstrated the ability to ensure the eventual capacity of the original connection [5,6]. A complete explanation of the optimisation methods and practical specificities can be found in [6].

### 2.3. Material Modelling

The optimisation process considered the case study’s web plate material, which was set to be A36 steel [17]. The mechanical parameters of this alloy are determined by a minimum yield strength of 250 MPa and a minimum ultimate strength of 400 MPa.

Nevertheless, other factors, such as information on the steel hardening phase and a reasonable level for ultimate elongation, were required to obtain truthful results from non-linear analysis. Consequently, the published literature was studied, including Sheikh-Ibrahim’s [7], Sheikh-Ibrahim and Frank’s [8], Mayatt’s [23], and Rex and Easterling’s [24] research, which suggested that it is reasonable to include a sensible and code-supported trilinear model with hardening. An elastic stage with E_0_ = 199.9 GPa, a plastic stage with E_0_/265, and a hardening stage with E_0_/1000 established such a model.

However, material models had to be changed to reflect actual steel characteristics when the chosen topology progressed to the production stage. The supplier’s material sheets confirmed the initial insight. BAMESA ensured a yield strength of 321 MPa, an ultimate strength of 434 MPa, and an elongation of 0.340 (mm/mm) for the steel batch from which the present plates were fabricated through subtractive manufacturing.

Nonetheless, steel coupons obtained from the steel plate have been tested for case-specific quantification. These results are provided in the next section and were utilised to obtain the updated trilinear model with hardening.

### 2.4. Manufactured Parts

#### 2.4.1. Steel Coupons

**Figure 2:**
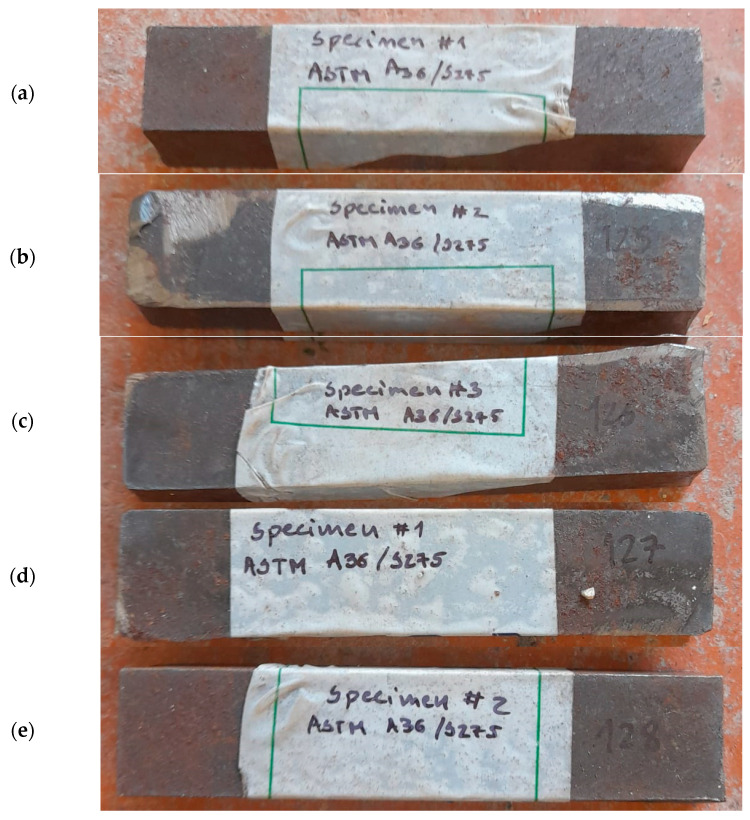
Steel coupons front and back: (**a**) Part 124, (**b**) Part 125, (**c**) Part 126, (**d**) Part 127, and (**e**) Part 128.

Material coupons, in the number of five, were obtained from the steel plate of which the prototypes were manufactured. These samples, listed in Table 1 and illustrated in Figure 2, were cut with a rectangular section and afterwards processed to meet the gauges prescribed in the EN 10025-2 [25] and ASTM A36 standards [17] for sample testing.

#### 2.4.2. Optimised Prototype

**Figure 3:**
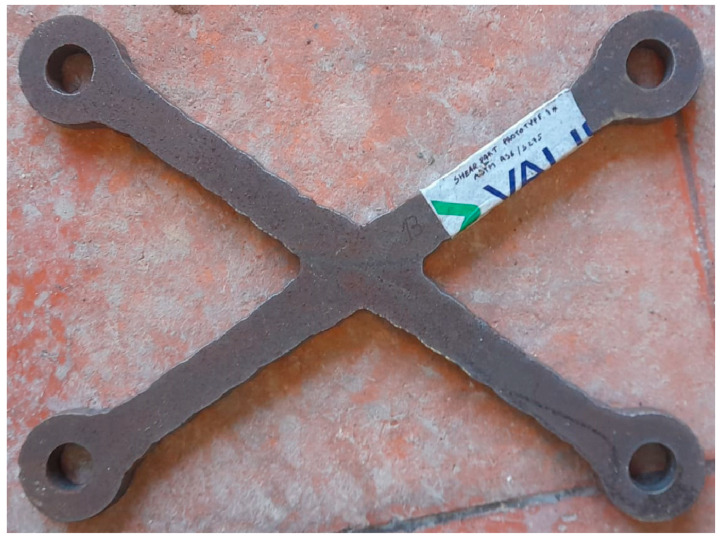
Optimised prototype.

The numerically optimised solution to a volume fraction of 12.5%—following the procedure depicted in [6]—was manufactured by VALIS, from a steel sheet with reference BAMESA lot 24474172FG and certificate 86.DO.01.22.A2, and was given the code “Part 13”. A photograph of the prototype is depicted in Figure 3.

## 3. Experimental Methods

### 3.1. Designing a Novel Testing Layout

**Figure 4:**
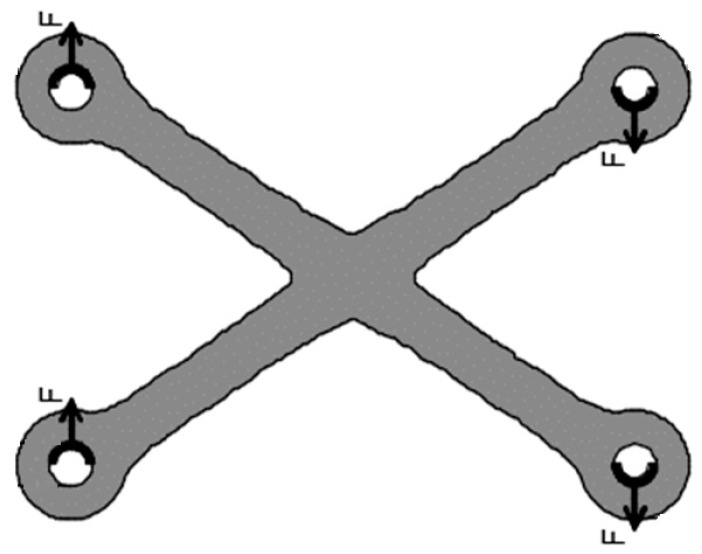
Shear plate loading.

Testing shear connections is a challenging laboratory endeavour since shear plates are loaded by bolts (Figure 4), which are not fixed. The plate will be subjected to a movement composed of translations and rotations, changing as the bolts experience different loading due to the movement, firstly, and due to the material non-linearity after. Therefore, fixed points within the plate domain are unlikely to exist.

Under these circumstances, testing layouts shall be designed to ensure the necessary degrees of freedom—thus avoiding unrealistic fixed points—and to obtain the loads applied by the correct elements with the correct intensities.

Therefore, the most reliable way to ensure these conditions is replicating the whole connection layout, including, most of the time, two shear cover plates, as well as the beam segments, a load actuator upon the latter, and fixed and sliding beam supports.

However, several problems arise from this approach. Using case-specific beam segments is expensive, time-consuming, and requires several different segments in each research programme. Moreover, loading control is indirect, as only the beam bending and shear moments at the splice can be commanded. The need to use two plates is equally a hindrance in most cases.

A testing layout has been designed to overcome these challenges to ensure a beam–splice-like interface assembled in a tension testing machine.

Made of easily removable and reassembled parts, this layout can be quickly assembled and disassembled. It is made of unique plates at the machine gripping sites, multiplied in the central part to ensure a symmetric loading configuration with double plates, to which a single shear testing plate can be bolted.

Hence, the plate behaviour can be simulated in real connection conditions (including accurate loading by bolts and plate movement) while the testing machine exerts direct control over load and displacement.

Contrary to indirect approaches, as in loading a beam with significant bending moments and shear forces, the machine-applied testing force equals the force to which the plate is subjected. Thus, it allows for the testing of much stronger plates with moderate laboratory resources.

**Figure 5:**
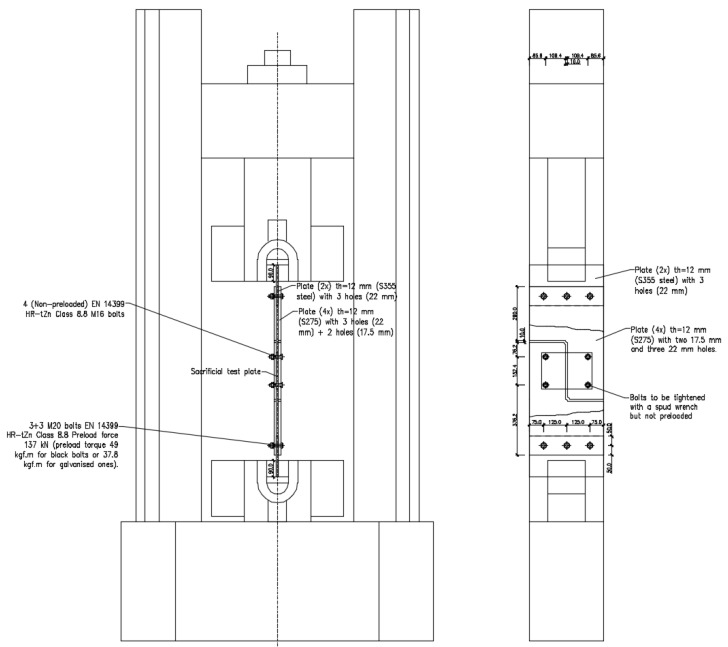
Testing layout for the sacrificial test plate.

**Figure 6:**
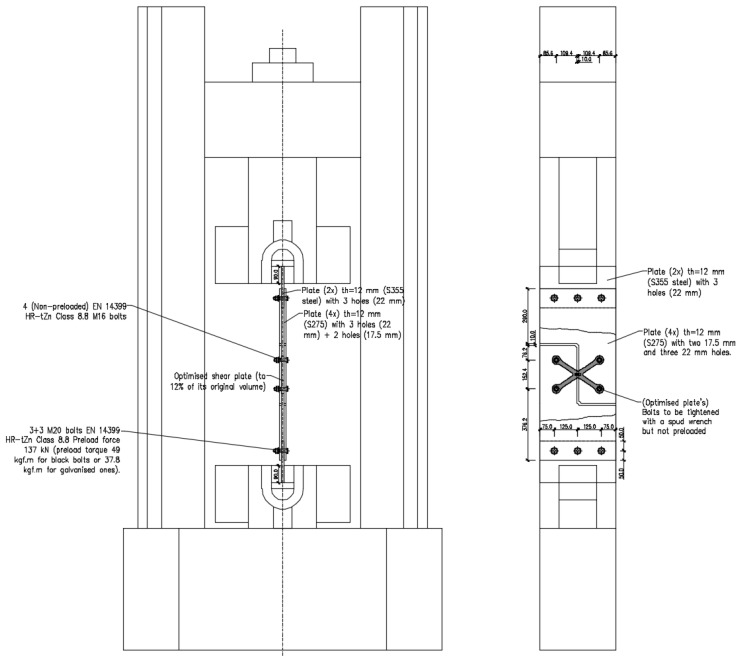
Testing layout for the topologically optimised steel plate.

The testing layout scheme can be found in Figure 5 for the sacrificial (non-optimised) plate and in Figure 6 for the topologically optimised plate.

### 3.2. Experimental Protocol

The experimental protocol may be divided into three steps: steel coupon testing for determining material properties, layout general rehearsal, and prototype testing.

Pertaining to the first step, the coupons’ test section is defined as a 12 mm × 6 mm section, and the parallel length is 50 mm, thus guaranteeing that the test results are admissible under both European (EN 10025-2) and American (ASTM A36) materials standards. To anchor the yielding position, a “*dog bone*” is required. The minimum number of tests required to generate characteristic values for assessing steel qualities has been determined to be three. Five specimens were created to confirm the test’s endurance to unanticipated circumstances, four of which were made and tested coupons.

**Figure 7:**
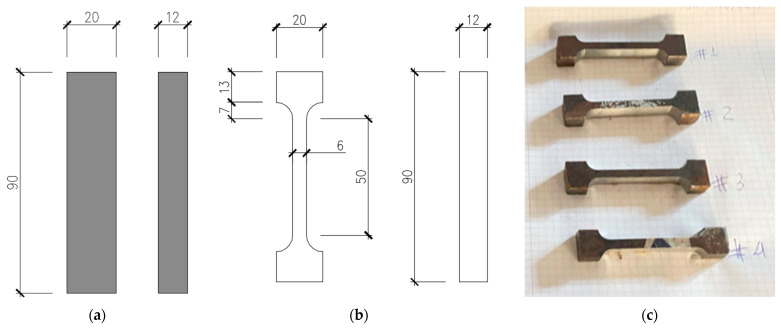
Steel coupon preparation and testing for material characterisation. (**a**) Manufactured specimens, (**b**) “*dog bone*” preparation, and (**c**) testing coupons.

The prismatic coupons were used to create four “*dog bone*” testing specimens. Figure 7 depicts the process used to prepare these specimens, and its purpose was to ensure 50 mm long testing gauges with a 12 mm × 6 mm section.

The coupons were tested until they failed in order to ascertain the steel’s yielding stress, ultimate stress, and ultimate strain. According to the literature on steel bolted connections [26,27], all tests were conducted in displacement control mode using a monotonic ramp with a 0.05 mm/min displacement rate.

**Figure 8:**
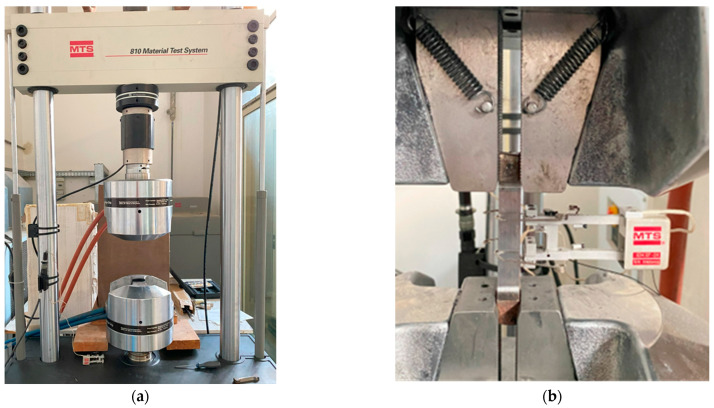
(**a**) Testing equipment used for the tensile tests on steel coupons and (**b**) a coupon under testing.

A universal testing machine, the “*MTS 810 Material Test System, model 318.25*” with a load capacity of 250 kN and clamping peak pressure of 69 MPa, was utilised for the tests. The strain within the specimens’ “*dog bone*” was measured both by crosshead motion, assuming uniform deformation along the 50 mm length of the reduced section (“*crosshead def*”), and by an integrated extensometer (“*MTS 634.12F-24, S/N 10183942E*”) with a gauge length of 25 mm (“*extensometer def*”). Figure 8 depicts an image of the testing machine.

**Figure 9:**
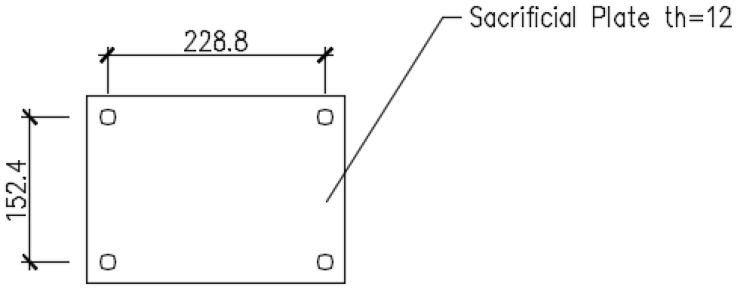
Sacrificial test plate.

In a second step, tests using “*sacrificial test plates*” were performed to evaluate the layout, measurement assembly, and testing conditions; to assess staff preparation; to inspect possible setup errors; and to determine the general conditions for following prototype testing. The “*sacrificial test plate*”, made of S275 steel to EN 10025, is displayed in Figure 9, and the testing configuration is shown in Figure 5. The bolts used to connect the testing plates to the layout were EN 14399’s HR-tZn class 8.8.

Once any adjustments deemed essential after evaluating the sacrificial plate testing results were applied, the prototype layout was gathered, as shown in Figure 6, and the tests then performed. According to the literature on steel bolted joints [26,27], the tests on the optimised shear prototypes were performed in displacement control mode considering a monotonic ramp and a 1 mm/min displacement rate.

**Figure 10:**
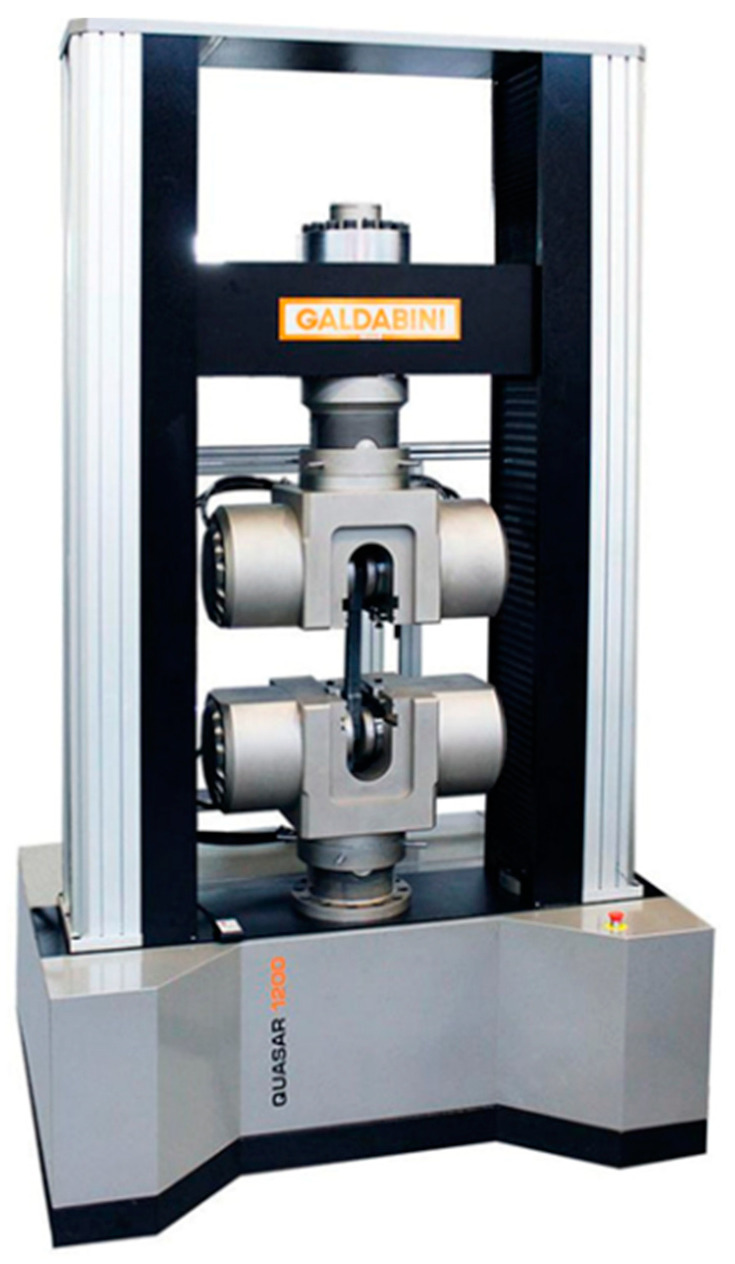
Testing machine used for the prototype tests.

A universal testing machine model, the “*Galdabini Quasar 1200 S/N VB47*” with 1200 kN of nominal load capacity, a stroke of 1300 mm, and a clamping force limit of 1800 kN, was employed for the tests. Figure 10 depicts the testing machine model.

**Figure 11:**
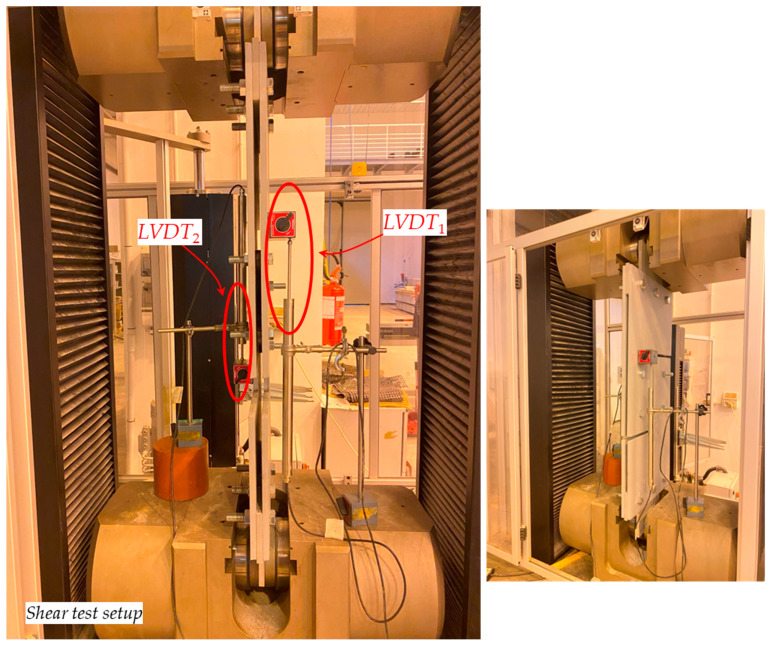
Configuration of LVDTs used for the tensile tests.

Beyond the vertical motion “*crosshead*” measurement at the testing frame, the local deformation of the prototype was computed using two linear variable displacement transducers (LVDTs) (model “*Monitran MTN/IEUSAL050-10*”, stroke ±50 mm, placed in a suitable configuration as shown in Figure 11 for the shear tests). The latter displacement measurement is referred to as “*LVDT*” and is the difference between the displacements detected by LVDT_1_ and LVDT_2_. The displacement measurement obtained via the two LVDTs is expected to represent the net displacement of the specimen. In contrast, the displacement measurement obtained through the crosshead motion shows the overall test setup’s gross displacement, which may be affected by possible slip phenomena at the wedges and further slip caused by bolt-hole clearance.

Both LVDT measurements were collected using an acquisition unit (model “*NI cDAQ 9189*”) that was outfitted with a suitable connection type (“*NI-9209*”) for monitoring input voltage signals. In the LabView environment, a personalised “project” was created to transform the voltage data into a displacement signal in real time and record signal data in a “.*txt*” file for postprocessing. All measurements (both those related to crosshead motion and those obtained via LVDTs) were taken at a sampling frequency of 10 Hz.

## 4. Results and Discussion

### 4.1. Tests for Material Properties Assessment

**Figure 12:**
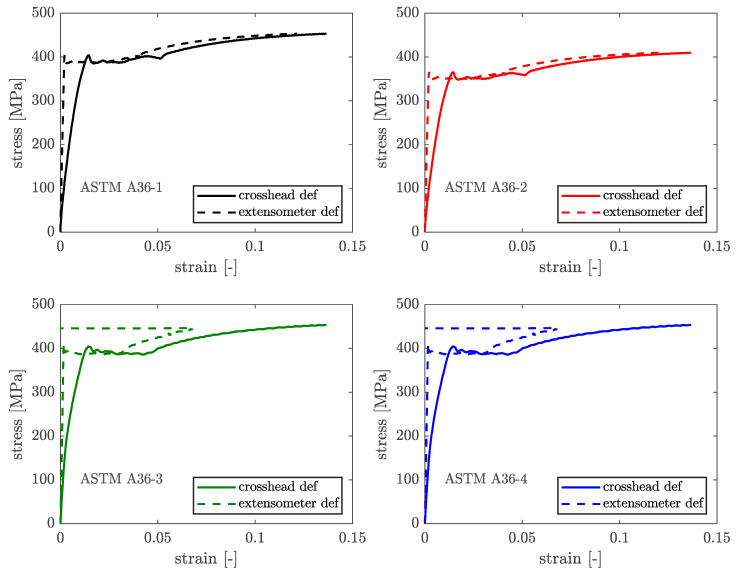
Stress–strain curves of four ASTM A36 coupons.

**Figure 13:**
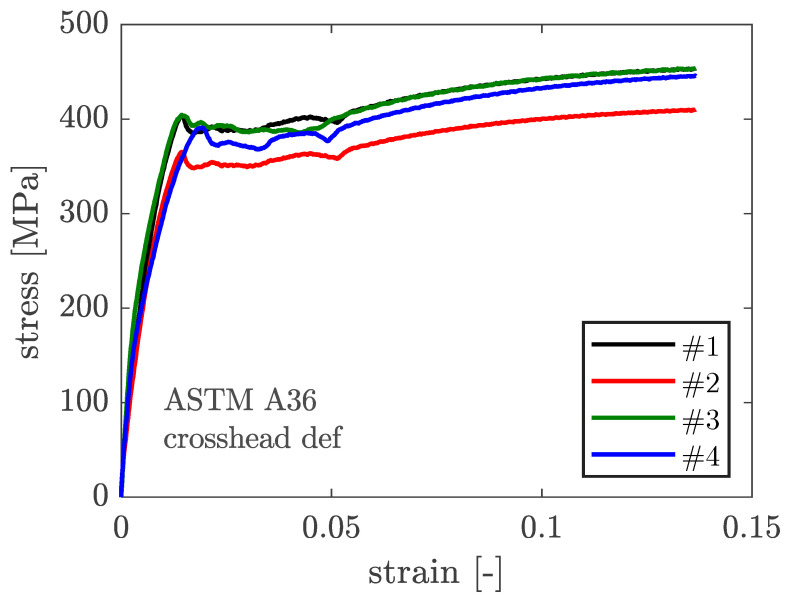
Contrast of the four stress–strain curves of ASTM A36.

Figure 12 depicts the attained stress–strain curves for the ASTM A36 steel coupons’ tensile tests considering crosshead deformation and extensometer deformation. Figure 13 compares the four stress–strain curves produced for the four specimens considering crosshead deformation. Table 2 shows the yielding and ultimate stress values for the four coupons, as well as the mean values and associated coefficient of variation (CoV).

As a result, the yield stress, which ASTM A36 limits to 250 MPa, has been reported in the material certificate as 321 MPa and was empirically determined as 397.9 MPa (CoV 2.05%). Furthermore, the ultimate stress, to which ASTM A36 prescribes no less than 400 MPa, is reported as 434 MPa in the material certificate and was empirically measured as 440.9 MPa (CoV 4.73%).

**Figure 14:**
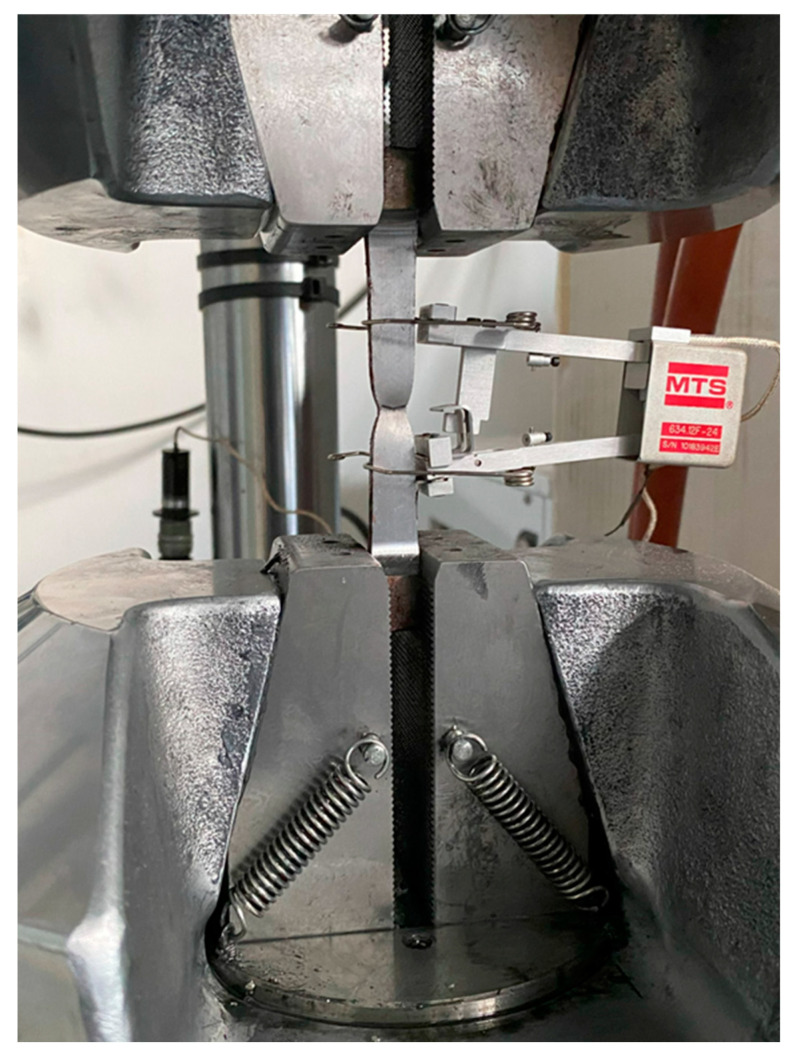
Failure of steel coupons made of ASTM A36.

The failure mechanism of the steel coupons happened in the middle section of the “*dog bone*” for all specimens, followed by a noticeable steel necking event, as shown in Figure 14.

**Figure 15:**
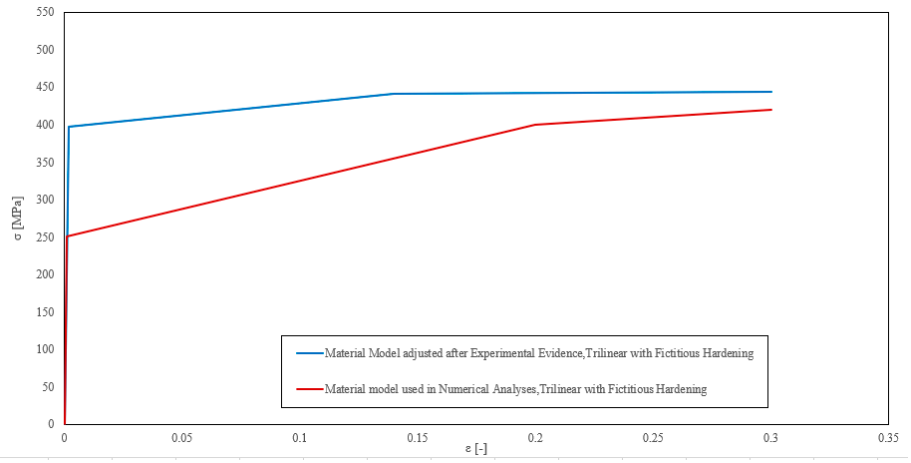
Stress–strain diagrams.

The stress–strain diagram was updated based on the experimental data, which were based on mathematical correlations and computational techniques [28,29,30,31]. Ultimate and yield thresholds were specified to the observed values, E_0_ was held constant at 199.9 GPa, and E was limited to E_0_/10,000 during the hardening phase to avoid a significant excess of the experimentally measured ultimate stress. The Young modulus of the plastic phase was set at E = E_0_/642. Figure 15 depicts both the original stress–strain diagram used in the non-linear analyses and the one modified in light of the experimental results.

### 4.2. Shear Test on the Sacrificial Plate

This preliminary trial aims to assess the correctness of the layout, geometry, general assumptions, and the non-optimised plate base scenario, including its failure mode, LVDT performance, installation and adequacy, and staff readiness for executing and recording tests.

Layout preparation was initiated by tightening the 3 + 3 M20 bolts at the extreme fixed plates by preloading them with the required torque (38 kgf.m), followed by manual tightening of the sacrificial test plate 2 + 2 M16 bolts with a spud wrench without preloading.

**Figure 16:**
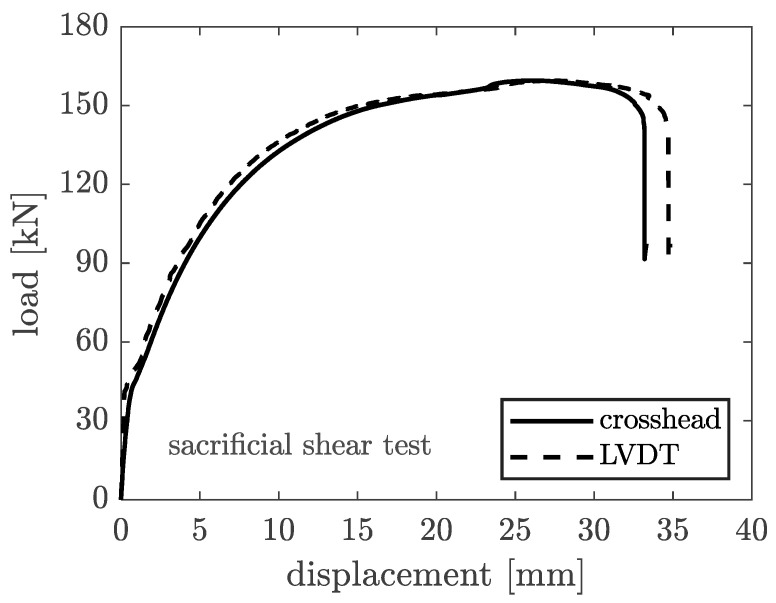
Load–displacement relation of the sacrificial test plate under shear.

Since the sacrificial plate for the shear test will not be used for subsequent tests, it was decided to perform the tests until complete collapse. The load–displacement relation for the sacrificial shear test plate is shown in Figure 16.

The prototype failed at a load of 159.5 kN, corresponding to displacement equal to 26.19 mm (measured through the crosshead motion) and 27.39 mm (measured through the LVDTs). The machine setup integrated crosshead motion and the LVDT measurements (acquired by the external cDAQ 9189 unit) which were relatively consistent up to the complete collapse of the specimen.

**Figure 17:**
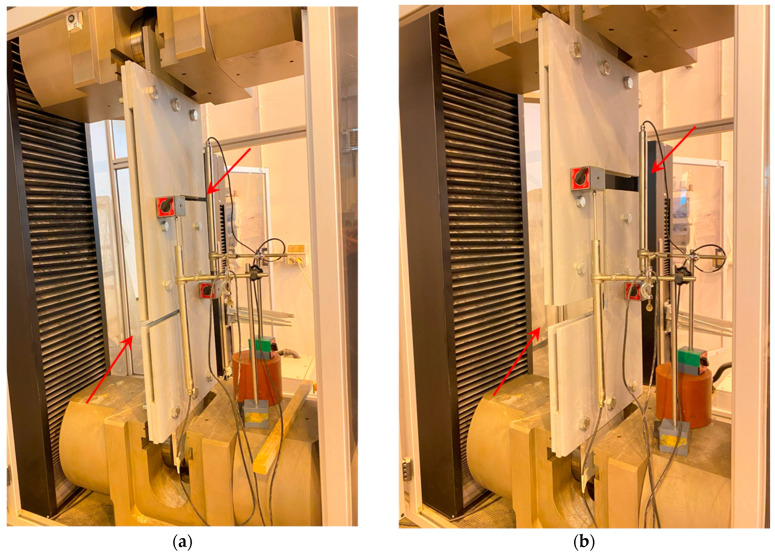
Shear test on the sacrificial plate: (**a**) beginning, (**b**) end of the test.

**Figure 18:**
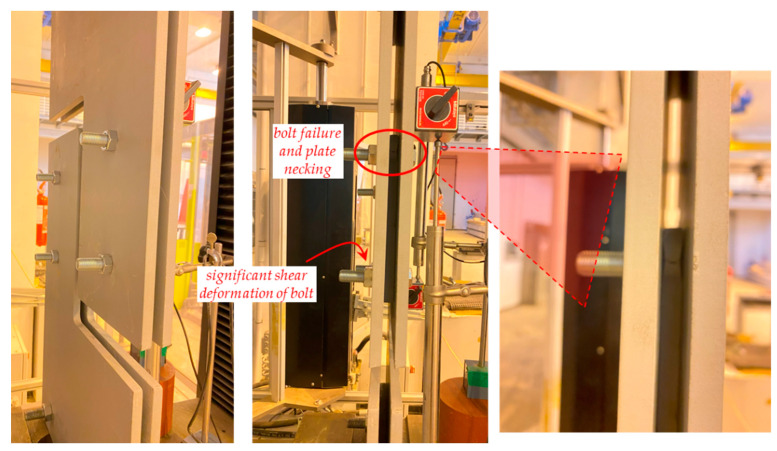
Failure of the sacrificial shear plate while in the testing machine.

**Figure 19:**
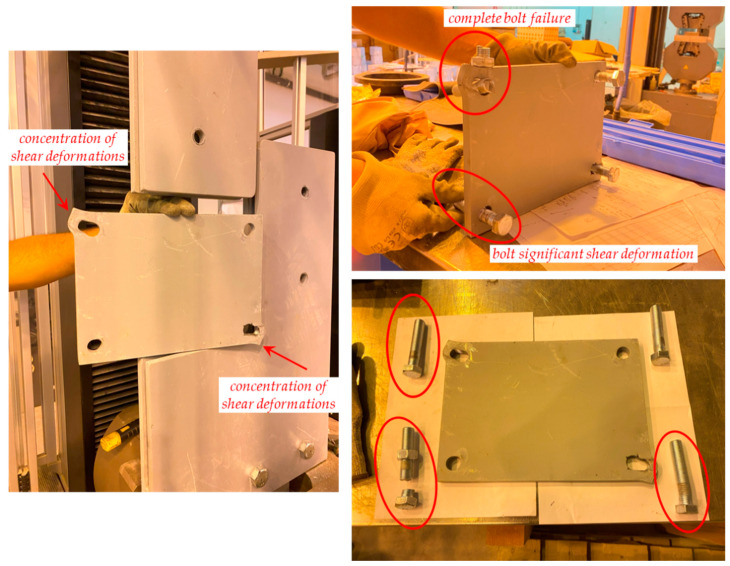
Failure of sacrificial shear plate extracted from the testing machine.

Some photographs of the shear test on the sacrificial plate are shown in Figure 17, while the failure of the prototype is shown in Figure 18 and Figure 19. In particular, the failure is ascribed to the bolt rupture of one of the four bolts, while significant shear deformations are observed in the other bolts along the bolt threaded area (especially in the bolt being vertically aligned with the former bolt that experienced the rupture). The two holes’ borders at the opposite corners of the sacrificial plate are markedly ovalised along the loading shear direction. The plate is considerably deformed, and a crack line develops around the bottom-right hole and propagates until the specimen edge along the horizontal direction (Figure 19).

It shall be noted that, despite the fact that neither the plate nor the bolt material has been tested to assess its actual capacity, failure occurring by bolt fracture due to shear is in line with the connection capacity calculations. In fact, failure was expected to occur at this mode when a total shear of 167 kN was applied to the plate. This test achieved a force of 159.5 kN before collapse, meaning a difference of approximately 4.5%.

### 4.3. Shear Test on the Optimised Prototype

A shear test was performed on specimen S1 (referred to as Part 13 within the manufacturing context). The 2 + 2 M16 bolts were tightened with a spud wrench without any applied preload, while preloading (38 kgf.m) was used on the 3 + 3 M20 bolts on plates fixed at the loading machine.

**Figure 20:**
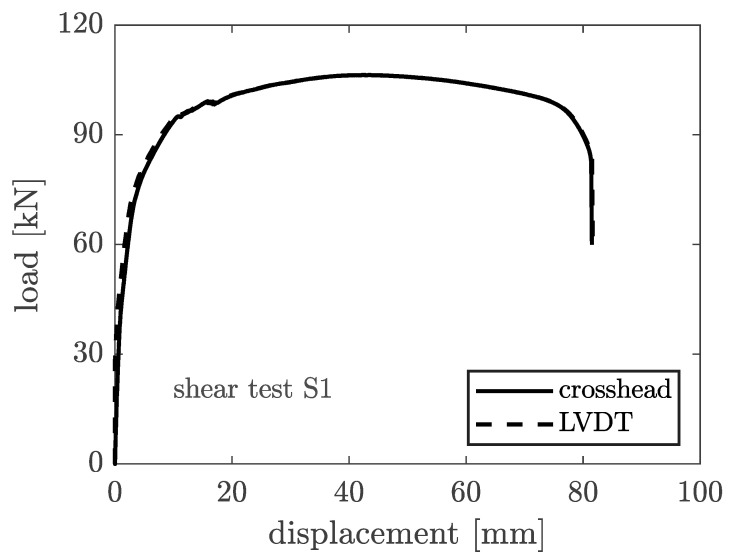
Load–displacement relation for the shear plate S1.

Figure 20 depicts the load–displacement curves obtained from the tested prototype (S1). The prototype failed with a load of 106.4 kN, equivalent to crosshead motion displacements of 43.62 mm and LVDT displacements of 43.53 mm. The two displacement measurements correspond remarkably, except for a minor difference in the linear elastic loading ramp of the curve. The prototype has shown significant ductility, with a slight post-peak softening. For a displacement of roughly 80 mm, the prototype experienced failure in shear.

**Figure 21:**
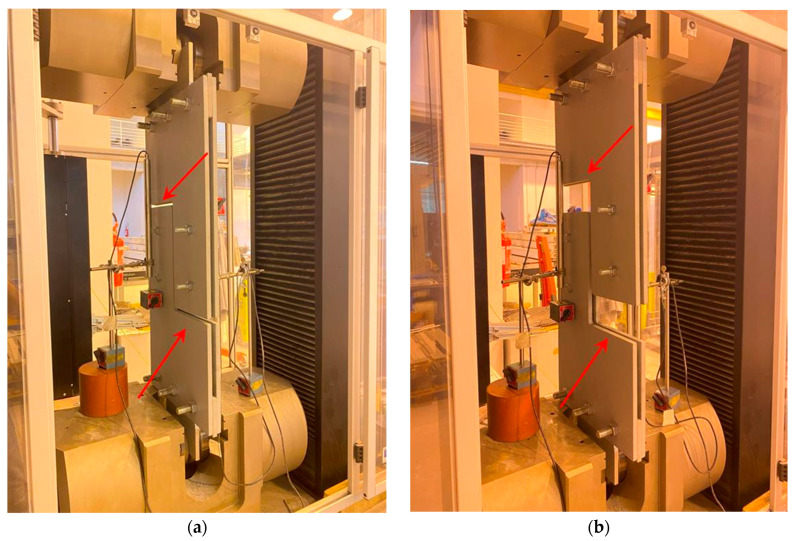
Shear test on the S1 plate: (**a**) beginning, (**b**) end of the test.

**Figure 22:**
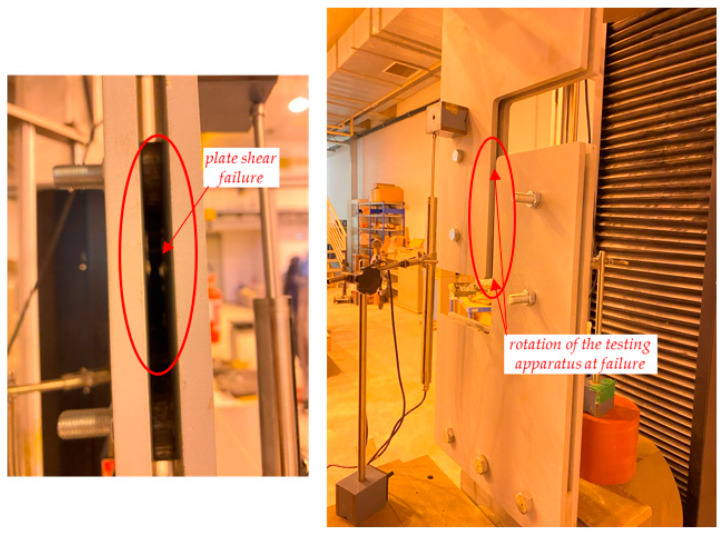
Failure of shear prototype S1 in the testing machine.

**Figure 23:**
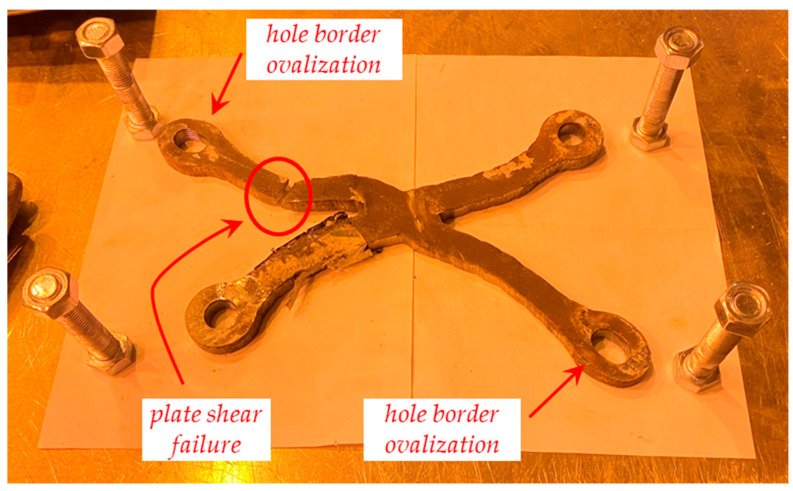
Failure of shear prototype S1 extracted from the testing machine.

Some photographs of the shear test on the S1 plate are shown in Figure 21, in which it is observed that the specimen exhibited a large shear deformation. The failure of the specimen is illustrated in Figure 22 and Figure 23. The former demonstrates the plate damage caused by shear loading while still assembled in the layout and the slight rotation of the layout plates, which can only be seen after significant vertical displacement. This, however, is a logical and expected phenomenon related to the degrees-of-freedom the layout allows to precisely match the conditions of the cover plate in a real connection. Furthermore, the collapse is ascribed to the plate shear failure of the upper-left segment of the X-shaped specimen, accompanied by significant hole border ovalisation observed in the two opposite corner bolts (top-left and bottom-right), which is consistent with the loading shear direction. No cracks are noted near the hole borders (Figure 23).

The ultimate capacity of the assessed prototype was experimentally found to be 106.4 kN. This outcome can be compared to the optimised plate’s adjusted ultimate capacity. Hence, by adjusting plate resistance to account for a thickness of 12.0 mm rather than 12.7 mm, as well as yield and ultimate stresses of 398 MPa and 441 MPa instead of the code-based values of 250 and 400 MPa, respectively, the prototype’s expected global ultimate characteristic resistance is 104.6 kN based on numerical analyses. Hence, one can find a difference of nearly 1.7% between the expected and the experimental ultimate capacity values.

## 5. Conclusions

A laboratory programme evaluated the behaviour of topologically optimised joint web plates and offered valuable information on the validity of prior computational physically non-linear analyses on those parts. The contributions can be summarized as follows:

- Tensile experiments were performed on coupons cut from the steel plate used to manufacture the optimised prototypes, matching European and American specifications for testing steel to structural standards for material properties definition.
- Experimental results fostered the development of a new trilinear material model, which will be useful for further numerical assessments in the optimised sections.
- A preliminary test to collapse, using the sacrificial test plate, was extremely useful for confirming the joint collapse mode—by bolt rupture—and the expected collapse load.
- The experimentally obtained ultimate capacity of the prototype closely matched the expected value. The former value not only slightly exceeded the numerical simulation findings, which are considered critical for attesting that the TO process is safe-sided for engineering design, but it also exceeded those values by roughly 1.7%, corroborating the numerical results.
- Future developments made possible with the herein attained results include reassessing numerical simulations, considering the experimentally defined steel properties, as well as expanding the research objects to other plates.

## Figures and Tables

**Table 1:** Steel coupons parts list.

Part	Type	Manuf.	Steel Supply	*f_y_* _(Stand_._)_	*f_u_*_(Stand_._)_	*f_y_*_(Certif_._)_	*f_u_*_(Certif_._)_	Certif.
124	Material Sample	Valis	BAMESA lot 24474172FG	250 MPa	400 MPa	321 MPa	434 MPa	86.DO.01.22.A2
125								
126								
127								
128								

**Table 2:** Tensile test results of coupons for yielding and ultimate stress.

Coupon	ASTM A36	
	*f_y_* [MPa]	*f_u_* [MPa]
124	404.0	453.6
125	404.0	410.1
126	397.1	454.1
127	386.7	445.9
Mean	**397.9**	**440.9**
CoV (%)	2.05	4.73

## Data Availability

Data are contained within the article.

## References

[1] Taha A.M., Dabaon M.A., El-Boghdadi M.H., Hassanein M.F. (2022). Experimental testing and evaluation of real-scale lap-splice bolted connections used in typical lattice steel transmission towers. Thin-Walled Struct..

[2] Kim J.S., Cho Y.H., Kim T.S. (2021). Recommendation on block shear strength equation of double shear four-bolted connection in cold-formed mild carbon steel. Structures.

[3] Yu J., Huang J., Li B., Feng X. (2021). Experimental study on steel plate shear walls with novel plate-frame connection. J. Constr. Steel Res..

[4] Zhao J., Wang Z., Peng Y., Liu X., Dong J. (2022). Experimental and numerical study on reduction coefficient of the shear capacity of austenitic long stainless-steel bolted connections. Eng. Struct..

[5] Ribeiro T., Bernardo L., Carrazedo R., De Domenico D. (2022). Eurocode-compliant Topology Optimisation and Analysis of a Steel Cover-plate in a Splice Moment Connection. Mater. Today Proc..

[6] Ribeiro T., Bernardo L., Carrazedo R., Domenico D. (2023). De Ultimate Capacity and Stability of Topologically Optimised Shear Plates in Compliance with Structural Eurocodes. Structures.

[7] Sheikh-Ibrahim F. (1995). Development of Design Procedures for Steel Girder Bolted Splices.

[8] Sheikh-Ibrahim F.I., Frank K.H. (2001). The ultimate strength of symmetric beam bolted splices. Eng. J..

[9] Richter C. (2017). Behavior of a Steel Girder Bolted Splice Connection.

[10] Ribeiro T., Bernardo L., Carrazedo R., De Domenico D. (2022). Eurocode-compliant topology optimisation of steel moment splice connections. J. Build. Eng..

[11] Ribeiro T., Bernardo L., Andrade J. (2021). Topology Optimisation in Structural Steel Design for Additive Manufacturing. Appl. Sci..

[12] Chen B., Roy K., Fang Z., Uzzaman A., Pham C.H., Raftery G.M., Lim J.B.P. (2022). Shear Capacity of Cold-Formed Steel Channels with Edge-Stiffened Web Holes, Unstiffened Web Holes, and Plain Webs. J. Struct. Eng..

[13] Milewska A., Roy K., Milewski P., Pham C.H., Charles Clifton G., Quenneville P., Lim J.B.P. (2022). Power-actuated fasteners in single shear-An experimental investigation and proposed design rules. Eng. Struct..

[14] Lu W., Ma Z., Mäkeläinen P., Outinen J. (2012). Behaviour of shear connectors in cold-formed steel sheeting at ambient and elevated temperatures. Thin Walled Struct..

[15] Fan L., Rondal J., Cescotto S. (1997). Finite element modelling of single lap screw connections in steel sheeting under static shear. Thin-Walled Struct..

[16] Cai K., Yuan H. (2023). Testing, numerical and analytical modelling of self-drilling screw connections between thin steel sheets in shear. Thin-Walled Struct..

[17] (2019). Standard Specification for Carbon Structural Steel 2019.

[18] (2005). Eurocode 3: Design of steel structures—Part 1–1: General rules and rules for buildings.

[19] (2005). Eurocode 3: Design of steel structures—Part 1–8: Design of joints.

[20] (2006). Eurocode 3—Design of Steel Structures—Part 1–5: Plated Structural Elements.

[21] Svanberg K. (1987). The method of moving asymptotes—A new method for structural optimization. Int. J. Numer. Methods Eng..

[22] Pedersen C.B.W., Allinger P. (2006). Industrial implementation and applications of topology optimization and future needs. Solid Mech. Appl..

[23] Adam J. (2012). Mayatt Structure-Property Relationships of an A36 Steel Alloy under Dynamic Loading Conditions.

[24] Rex C., Samuel Easterling W. (1996). Behavior and Modeling of Mild and Reinforcing Steel.

[25] (2005). Hot rolled products of structural steels - Part 2: Technical delivery conditions for non-alloy structural steels.

[26] Jiang K., Zhao O., Tan K.H. (2020). Experimental and numerical study of S700 high strength steel double shear bolted connections in tension. Eng. Struct..

[27] Jiang K., Tan K.H., Zhao O., Gardner L. (2021). Block tearing of S700 high strength steel bolted connections: Testing, numerical modelling and design. Eng. Struct..

[28] Cheng H., Paz C.M., Pinheiro B.C., Estefen S.F. (2020). Experimentally based parameters applied to concrete damage plasticity model for strain hardening cementitious composite in sandwich pipes. Mater. Struct. Constr..

[29] Zakir Sarothi S., Sakil Ahmed K., Imtiaz Khan N., Ahmed A., Nehdi M.L. (2022). Machine learning-based failure mode identification of double shear bolted connections in structural steel. Eng. Fail. Anal..

[30] Sousa A.M., Matos H.A., Pereira M.J. (2022). Properties of Crude Oil-in-Water and Water-in-Crude Oil Emulsions: A Critical Review. Ind. Eng. Chem. Res..

[31] Kořínek M., Halama R., Fojtík F., Pagáč M., Krček J., Krzikalla D., Kocich R., Kunčická L. (2021). Monotonic tension-torsion experiments and fe modeling on notched specimens produced by slm technology from ss316l. Materials.

